# Identification and Molecular Binding Mechanism of Novel α-Glucosidase Inhibitory Peptides from Hot-Pressed Peanut Meal Protein Hydrolysates

**DOI:** 10.3390/foods12030663

**Published:** 2023-02-03

**Authors:** Xinyu Yang, Dan Wang, Yangyong Dai, Luping Zhao, Wentao Wang, Xiuzhen Ding

**Affiliations:** Engineering and Technology Center for Grain Processing of Shandong Province, Key Laboratory of Food Processing Technology and Quality Control in Shandong Province, College of Food Science and Engineering, Shandong Agricultural University, 61 Daizong Avenue, Taian 271018, China

**Keywords:** hot-pressed peanut meal protein, α-glucosidase inhibition, molecular docking, molecular mechanism, stability

## Abstract

Hot-pressed peanut meal protein hydrolysates are rich in Arg residue, but there is a lack of research on their α-glucosidase inhibitory activity. In this study, different proteases were used to produce hot-pressed peanut meal protein hydrolysates (PMHs) to evaluate the α-glucosidase inhibitory activity. All PMHs showed good α-glucosidase inhibitory activity with the best inhibition effect coming from the dual enzyme system of Alcalase and Neutrase with an IC_50_ of 5.63 ± 0.19 mg/mL. The fractions with the highest inhibition effect were separated and purified using ultrafiltration and cation exchange chromatography. Four novel α-glucosidase inhibitory peptides (FYNPAAGR, PGVLPVAS, FFVPPSQQ, and FSYNPQAG) were identified by nano-HPLC-MS/MS and molecular docking. Molecular docking showed that peptides could occupy the active pocket of α-glucosidase through hydrogen bonding, hydrophobic interaction, salt bridges, and π-stacking, thus preventing the formation of complexes between α-glucosidase and the substrate. In addition, the α-glucosidase inhibitory activity of PMHs was stable against hot, pH treatment and in vitro gastrointestinal digestion. The study demonstrated that PMHs might be used as a natural anti-diabetic material with the potential to inhibit α-glucosidase.

## 1. Introduction

Diabetes mellitus (DM) is an endocrine and metabolic disease characterized by hyperglycemia worldwide. As reported in the 2019 International Diabetes Federation (IDF) atlas, 463 million adults between the ages of 20 and 79 have DM, and this number is expected to rise to 702.2 million by 2045. Ninety percent of DM is Type 2 diabetes mellitus (T2DM) [1]. One of the promising strategies for combating T2DM is to slow down the release of glucose into the bloodstream by inhibiting the carbohydrate digestion relating enzymes, such as α-glucosidase [2]. Acarbose, voglibose, and miglitol are well-recognized drugs that can control blood sugar levels, most of which are chemical synthetic drugs. In addition, long-term usage will be accompanied by a series of side effects such as abdominal distension, diarrhea, and abdominal pain [3,4]. Therefore, it is pressing to explore novel and safe drugs derived from natural products in the treatment of T2DM. In recent years, more and more attention has been paid to the extraction of α-glucosidase inhibitory peptides from safe natural products, and a variety of α-glucosidase inhibitory peptides have been isolated from food proteins, such as soybean, wheat germ, buffalo milk, quinoa, silkworm [5,6,7,8,9].

Peanut meals are by-products of the peanut oil processing industry [10]. Peanut oil extracted by hot-press is an important method of peanut processing in China, and a lot of hot-pressed peanut meals are yearly produced. The high-quality protein content of peanut meal is only second to soybean meals and hot-pressed peanut meals are considered to be one of the most promising plant protein sources [11]. However, due to the low solubility of hot-press peanut meal protein, poor functional properties, low utilization rate, and economic benefits [12,13], peanut meals are mainly used as feed, fertilizer, and brewing raw materials. Recently, research showed protease hydrolysis could hydrolysate insoluble peanut meal protein into small soluble peptides. The advantages of mild conditions, green energy saving, good product quality, and broad application prospects [14], make protease hydrolysis become the best process for protein extraction in peanut meals. Meanwhile, peanut protein hydrolysates exhibited many bioactivities, such as antioxidant, reducibility, angiotensin I-converting enzyme inhibitory activity, and hypotensive properties [14,15,16,17,18]. Interestingly, peanut meal proteins are rich in arginine, one of the features of α-glucosidase inhibitory peptides. To our knowledge, to date, there is very little research regarding the isolation and identification of α-glucosidase inhibition in hot-pressed peanut meal hydrolysates (PMHs), and little is known about the characteristic and α-glucosidase inhibitory mechanism of PMHs.

Therefore, in this research, we explored the influence of various proteases on the α-glucosidase inhibitory activity of PMHs and selected the enzyme with the strongest inhibitory activity to prepare α-glucosidase inhibitory peptides. Peptides with the best α-glucosidase inhibitory were isolated by ultrafiltration and ion exchange chromatography and further identified by nano-HPLC-MS/MS. Possible α-glucosidase inhibition mechanisms of PMHs were investigated by molecular docking. At last, the inhibitory stability of PMHs against pH, hot, and in vitro gastrointestinal digestion was evaluated.

## 2. Materials and Methods

### 2.1. Materials

Defatted hot-pressed peanut meals were gifts of Shandong Jinsheng Grain Oil and Food Co., Ltd. (Linyi, China). Alcalase, Protamex, Papain, Flavourzyme, and Neutrase were purchased from Novozymes (Beijing, China). α-glucosidase and its substrate p-nitrophenyl-α-D-glucopyranoside (pNPG) were bought from Shanghai Yuanye Biotechnology Co., Ltd. (Shanghai, China). Other reagents were analytically pure.

### 2.2. Preparation of PMHs

The PMHs were produced by Alcalase (pH 8.0, 50 °C), Protamex (pH 7.5, 50 °C), Neutrase (pH 7.0, 50 °C), Flavourzyme (pH 7.5, 55 °C), Papain (pH 7.0, 55 °C) and a double enzyme system (Neutrase and Alcalase), respectively. The peanut meals were added into deionized water with a concentration of 10% and stirred for 30 min. Then, the pH and temperature of the dispersions were adjusted to the optimum working conditions of each enzyme. One percent of the protease was added to initiate the hydrolysis which lasted for 4 h. The hydrolysis time was 2 h for each protease in the double enzyme system. The proteases were inactivated by boiling for 15 min. After adjusting to pH 7.0, the hydrolysates were centrifuged for 20 min at 4000 rpm. The resulting supernatant was freeze-dried and stored at −20 °C until use.

### 2.3. Determination of Degree of Hydrolysis

The degree of hydrolysis (DH) was measured by the pH-stat method as described by Rios-Herrera et al. [19] with the *h_tot_* recognized as 7.55 meqv/g.

### 2.4. Determination of Trichloroacetic Acid-Nitrogen Soluble Index (TCA-NSI)

The TCA-NSI was determined according to the method of Kong [20] with minor modifications. The enzymatic hydrolysate solution was pipetted to 15% TCA solution with the same volume and mixed, stood for 20 min, and then centrifuged for 20 min (5000 rpm) at ambient temperature. The nitrogen content in the supernatant and the initial hydrolysate solution was determined by the Kjeldahl method. The calculation formula of TCA-NSI was as follows:

(1) $$TCA-NSI\;\left(\%\right)=\left(\frac{N}{N_{0}}\right)\times 100$$

where *N* is the nitrogen content of the supernatant; *N*_0_ is the nitrogen content of the initial hydrolysate solution.

### 2.5. Determination of Protein Yield

The nitrogen yields of the hydrolysis were calculated as follows:

(2) $$Yield\;\left(\%\right)=\left(\frac{N_{h}}{N_{m}}\right)\times 100$$

where *N_h_* is the nitrogen content of the hydrolysates (g) and *N_m_* is the nitrogen content in the hot-pressed peanut meals (g).

### 2.6. Determination of α-Glucosidase Inhibitory Activity

The determination method of α-glucosidase inhibitory activity described by previous studies [21,22,23] was used with modifications. An amount of 20 μL of 0.01 M PBS (pH 6.8), 20 μL of 0.3 U/mL α-glucosidase, and 20 μL of PMHs were pipetted into a well of the 96-well plate and mixed. After the mixture was incubated at 37 °C for 15 min, 20 μL of 2.5 mM pNPG was added to start the reaction. After 30 min at 37 °C, 80 μL Na_2_CO_3_ (0.2 M) was added to terminate the reaction. The absorbance was detected and recorded at 405 nm using a microplate reader. In the sample blank, the same amount of PBS was used to replace α-glucosidase; in the blank control the same amount of PBS was used to replace the hydrolysate solution, and only PBS and Na_2_CO_3_ were added to the blank. The α-glucosidase inhibitory rate was calculated as follows:

(3) $$\begin{array}{c} \alpha \text{-}\mathrm{glucosidase}\;\mathrm{inhibitory}\;\mathrm{rate}\;\left(\%\right)=\left[1-\frac{A_{sample\;reaction}{-A}_{sample\;blank}}{A_{blank\;control}{-A}_{blank}}\right]\times 100 \end{array}$$

### 2.7. Determination of Molecular Weight Distribution

The molecular weight distribution of PMHs was determined using size exclusion high-performance liquid chromatography (SEC-HPLC). The detection conditions were as follows: the chromatographic column being TSK gel G2000 SW_XL_ (7.8 × 300 mm), eluate being ultra-pure water: acetonitrile: Trifluoroacetic acid = 55:45:0.1 (*v*/*v*/*v*), detection wavelength is 220 nm, flow rate being 0.5 mL/min. The temperature of the column was controlled at 30 °C.

### 2.8. Separation of α-Glucosidase Inhibitory Peptides from PMHs

#### 2.8.1. Ultrafiltration

The PMHs with strongest activity were separated by ultrafiltration membranes with molecular weight cut-offs (MWCO) of 3 kDa and 10 kDa, respectively. Three fractions with molecular weights <3 kDa, 3~10 kDa, and >10 kDa were collected and lyophilized. The inhibitory rate against α-glucosidase was determined and IC_50_ was calculated.

#### 2.8.2. Ion Exchange Chromatography

The ultrafiltration fraction with the strongest α-glucosidase inhibitory activity was dissolved in deionized water (20 mg/mL) and further separated via a 732-cation exchange resin column (26 × 300 mm) equilibrated with deionized water. The loading quality was 5 mL. The column was eluted with different concentrations of ammoniac solutions (0, 5, 7.5, 10, and 15%) at a flow rate of 4 mL/min, and the eluate was detected at 220 nm. The peaks of the eluates were collected, concentrated, dialyzed, and freeze-dried. The α-glucosidase inhibitory rate of each separated fraction was determined at a concentration of 0.27 mg/mL.

### 2.9. Identification of α-Glucosidase Inhibitory Peptides Using Nano-HPLC-MS/MS

The separated fraction with the strongest α-glucosidase inhibitory activity was identified. A nano upgraded high-performance liquid chromatography (EASY-nLC 1200, Thermo Fisher Scientific, Waltham, MA, USA) equipped with a C18 column (50 μm × 150 mm, Acclaim PepMap^TM^ RSLC, Thermo Fisher Scientific, Waltham, MA, USA) and high-resolution mass spectrometry (Q-Exactive HF, Thermo Fisher Scientific, Waltham, MA, USA) was employed. Desalting was performed using a Waters SEP-PAK C18 solid phase extraction column. The desalted sample was dissolved in 0.1% (*v*/*v*) formic acid solution to a concentration of 1 mg/mL. The injection volume was 1.0 μL. The flow rate was 300 nL/min, and the column temperature was 30 °C. Mobile phase A was ultrapure water with 0.1% (*v*/*v*) formic acid, and mobile phase B was acetonitrile with 0.1% (*v*/*v*) formic acid. The chromatographic separation was carried out as follows: 4–28% B (0–98 min); 28–37% B (98–113 min); 37–100% B (113–117 min); 100% B (117–120 min). The mass spectral conditions were as follows: (1) detection mode: positive ions; (2) MS: scan rage (*m*/*z*) = 400–1800; resolution = 60,000; automatic gain control (AGC) target = 3 × 10^6^; maximum injection time 50 ms; (3) MS/MS: resolution = 15,000; isolation window = 1.6; AGC target = 2 × 10^5^; maximum injection time = 20 ms; (4) collision energy = 28 eV.

### 2.10. Molecular Docking Analysis

2QMJ, the crystal structure of α-glucosidase, came from the Research Collaboratory for Structural Biology Protein Data Bank (https://www.rcsb.org/structure/2QMJ, accessed on 1 November 2022) [7]. Chem-Bio3D Ultra 20.0 package was applied to predict the 2D structures and convert the 3D structures of the peptides. AutoDock Tools 1.5.7 package was employed to create the input files for docking. Autodock vina 1.2.2 was applied to simulate the action between peptides and α-glucosidase with flexible docking. The output poses were evaluated by the Gird scoring function, with the lowest score recognized as the predicted action mode [24]. The search grid of the α-glucosidase was set as center x: −21.56, center y: −6.936, and center z: −1.971 with the grid spacing of docking parameters set to 0.375 Å and the size set 60 Å × 60 Å × 60 Å. The value of exhaustiveness was 20. The binding sites were visualized using PyMol 2.6 (http://www.pymol.org/, accessed on 18 August 2022).

### 2.11. Stability of α-Glucosidase Inhibitory Peptides

The determination of inhibitory stability against α-glucosidase of PMHs under pH, hot, and in vitro gastrointestinal digestion was modified according to the method of Ketnawa et al. [25] and Brodkorb et al. [26]. In order to explore the thermal stability of peptides, 10 mL of peptides (5 mg/mL) was placed at 37, 50, 60, 70, 80, 90, 100, and 121 °C, respectively. Then, the α-glucosidase inhibitory rate was determined. To explore the stability against pH, the peptide solutions (10 mg/mL) were treated at pH 1.0, 3.0, 5.0, 7.0, 9.0, 11.0, or 13.0 for 30 min at the ambient temperature, after which the α-glucosidase inhibitory rate was determined. For stability during in vitro gastrointestinal digestion, the PMHs solution (10 mg/mL) was mixed with simulated saliva (SSF) and stood for 2 min at 37 °C and pH 7. Then, the peptide solution was mixed with simulated gastric digestive juice (SGF) and incubated for 120 min at 37 °C and pH 2. Finally, the peptide mixture was mixed with simulated intestinal digestive juice (SIF) and bile salt and incubated for at 37 °C and pH 7.5. The samples at 2, 32, 62, 92, 122, 152, 182, 212, and 242 min were collected and inactivated. At last, the α-glucosidase inhibitory rate was determined. The α-glucosidase inhibitory stability was expressed as activity relative to that of PMHs with no treatment (100%).

### 2.12. Statistical Analysis

All the tests were carried out at least three times, and data were presented as mean values ± standard deviation. The significance was analyzed by one-way analysis of variance (ANOVA) through SPSS 25.0 (IBM, Armonk, NY, USA).

## 3. Results and Discussion

### 3.1. Degree of Hydrolysis and α-Glucosidase Inhibitory Activity of PMHs

**Figure 1:**
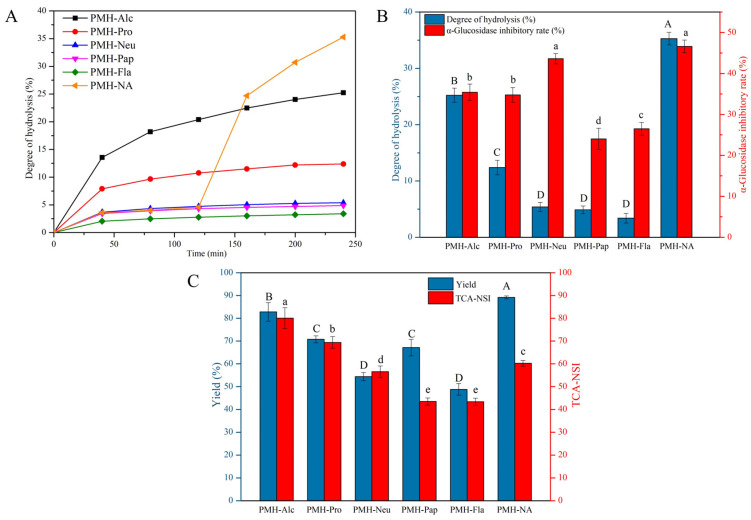
(**A**) Degree of hydrolysis during the hydrolysis of different hydrolysates. (**B**) Nitrogen solubility index and protein yield of different hydrolysates. (**C**) Comparison of degree of hydrolysis and α-glucosidase inhibition rate of different hydrolysates. Different letters indicate significant differences (*p* < 0.05).

Each enzyme has its specific catalytic site(s) for the hydrolysis of proteins, releasing peptides with different molecular weights, amino acid sequences, and bioactivities [27]. To evaluate the ability of five commercial proteases on the release of α-glucosidase inhibitory peptides from hot-pressed peanut meal proteins, hydrolysis was performed and DH, protein yield, TCA-NSI, and α-glucosidase inhibitory rate were determined. The plots of DH versus time are shown in Figure 1A, which increased with time. As expected, the hydrolysis rate increased rapidly at first, and then decreased gradually. It was probably due to the adequate contact between the enzyme and the substrate leading to rapid hydrolysis at the initial stage. As the reaction proceeded, the contact between the enzyme and the substrate became small resulting in a slowed-down hydrolysis [28]. Alternatively, the hydrolysis rate may be reduced due to the inhibition of the enzyme or the denaturation of the enzyme The tendency was similar to the hydrolysis of silkworm pupae protein and camellia seed protein [29,30]. The final DH of different enzyme hydrolysates is shown in Figure 1B. The hydrolysates of Alcalase, Protamex, Neutrase, Papain, and Flavourzyme were represented as PMH-Alc, PMH-Pro, PMH-Neu, PMH-Pap, and PMH-Fla, respectively. PMH-Alc exhibited the highest DH, followed by PMH-Pro, PMH-Neu, PMH-Pap, and PMH-Fla. This indicated that Alcalase had the strongest hydrolysis ability toward hot-pressed peanut meal protein. Kong et al. [20] chose Alcalase to hydrolyze walnut proteins and Wang et al. [31] hydrolyzed peony seed proteins with Alcalase, both of which exhibited high hydrolytic capacity. This might be attributed that Alcalase was an endoprotease and had more catalytic sites to sever the peptide bonds in the protein, allowing for deeper enzymatic hydrolysis [32]. Meanwhile, PMH-Alc showed the highest protein yield, and PMH-Neu showed the lowest, indicating that the protein yield was not only associated with DH but also the solubility of peptides released. As shown in Figure 1C, the highest TCA-NSI value came from PMH-Alc (80.06 ± 4.54%), followed by PMH-Pro (69.36 ± 2.54%), PMH-Neu (56.53 ± 2.56%), PMH-Pap (43.48 ± 1.54%) and PMH-Fla (43.41 ± 1.57%). The TCA-NSI values were positively correlated with DH. As for α-glucosidase inhibitory activity, PMH-Neu showed the strongest activity, followed by PMH-Alc, PMH-Pro, PMH-Fla, and PMH-Pap.

Although PMH-Neu showed the strongest α-glucosidase inhibitory activity, DH, yield, and TCA-NSI were significantly smaller (*p* < 0.05) than that of PMH-Alc. Therefore, a double enzyme system of Neutrase and Alcalase was further applied to increase the DH, yield and TCA-NSI. The resulting hydrolysate was designated as PMH-NA. Compared with the single-enzyme system, the dual-enzyme system significantly increased the yield and DH. As shown in Figure 1, the DH and protein yield of PMH-NA were significantly higher (*p* < 0.05) than that of PMH-Alc and PMH-Neu. Although the TCA-NSI of PMH-NA was smaller than that of PMH-Alc, it was significantly higher (*p* < 0.05) than that of PMH-Neu. PMH-NA showed similar α-glucosidase inhibitory activity to PMH-Neu, with an IC_50_ value of 5.63 ± 0.19 mg/mL. This IC_50_ value was lower than that of Ginkgo biloba seed protein hydrolysate produced by Alcalase with an IC_50_ value of 12.94 ± 0.37 mg/mL [33] and black cricket protein hydrolysates produced by a combination of Neutrase and Flavourzyme with an IC_50_ value of 6.21 ± 0.41 mg/mL [34]. This suggested that PMH-NA was a promising natural component for the regulation of diabetes. Yan et al. [35] reported that hydrolysis of rice proteins using a dual-enzyme system resulted in an increased concentration of small peptides, reduced bitterness, and improved hydrolysis compared to protease alone. Cian et al. [36] used a dual-enzyme system to prepare hydrolysates from Porphyra columbina by-products, demonstrating higher levels of hydrolysis and small peptide content than one enzyme alone.

### 3.2. Isolation and Purification of α-Glucosidase Inhibitory Peptide

**Figure 2:**
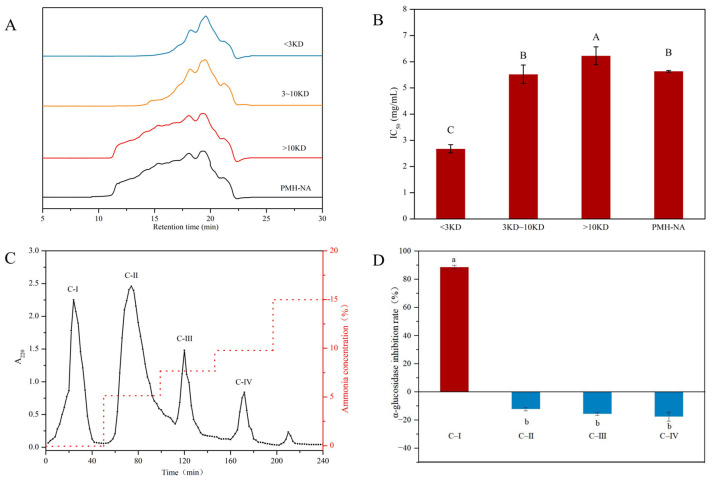
(**A**) SEC-HPLC profiles of fractions after ultrafiltration. (**B**) IC_50_ value of each fraction after ultrafiltration. (**C**) Elution spectra of <3 kDa components on cation exchange column. (**D**) α-glucosidase inhibition rate of fractions separated by cation exchange column. Different letters indicate significant differences (*p* < 0.05).

Peptides in PMH-NA were further separated and purified by ultrafiltration and ion-exchange chromatography. The MWCOs of ultrafiltration were 3 kDa and 10 kDa, and three fractions of >10 kDa, 3–10 kDa, and <3 kDa were obtained. Figure 2A presents the SEC-HPLC profiles of each fraction, while Figure 2B shows the α-glucosidase inhibitory activity. It was obvious that the molecular weight of fraction <3 kDa was lower than the other two fractions. In addition, compared to the other two fractions, the fraction of <3 kDa had the lowest IC_50_ and exhibited the most potent α-glucosidase inhibitory activity, indicating small peptides possessed stronger α-glucosidase inhibitory potential. Consistently, Zhao et al. [9] also found that the buffalo milk protein hydrolysates of less than 2000 Da showed stronger α-glucosidase inhibitory activity than fractions with higher size. Agrawal et al. [37] showed in their study that peptides with low molecular weights were more likely to react with α-glucosidase inhibitory. Meanwhile, for long peptides and high molecular weight peptides, the binding to α-glucosidase active sites was spatially blocked and cannot effectively bind to α-glucosidase active sites.

The fraction of <3 kDa was then further separated by ion exchange chromatography. Four fractions (C-I, C-II, C-III, C-IV) were collected and desalted with a 100 Da dialysis membrane. The α-glucosidase inhibitory rate at a concentration of 0.27 mg/mL is shown in Figure 2D. It was interesting that C-I showed inhibitory activity on α-glucosidase, while C-II, C-III, and C-IV promoted the activity of α-glucosidase, this may be due to some peptides that promote the binding between α-glucosidase and to the substrate. In a previous study, Wang et al. [38] used DEAE-52 ion exchange resin to purify soybean peptides to obtain fractions with high α-glucosidase inhibition. It was found that only the fraction eluted with distilled water had α-glucosidase inhibition activity and the inhibition activity was much higher than that of the original hydrolysate. The α-glucosidase inhibitory rate of C-I was 88.75 ± 1.23% with an IC_50_ value of 0.185 ± 0.034 mg/mL. The activity was 30.40-fold of PMHs before separation and purification. Therefore, C-I was chosen for subsequent analysis.

### 3.3. Identification and Screening of α-Glucosidase Inhibitory Peptides in C-I Fraction

**Figure 3:**
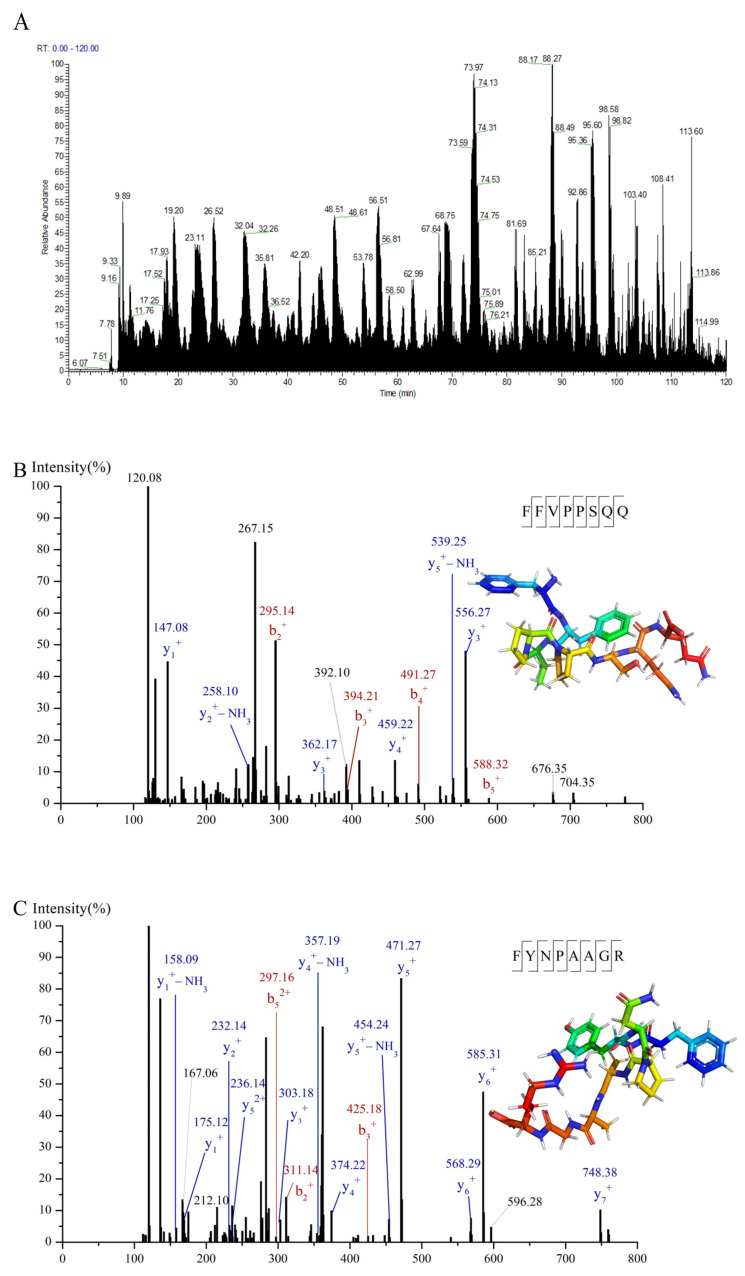

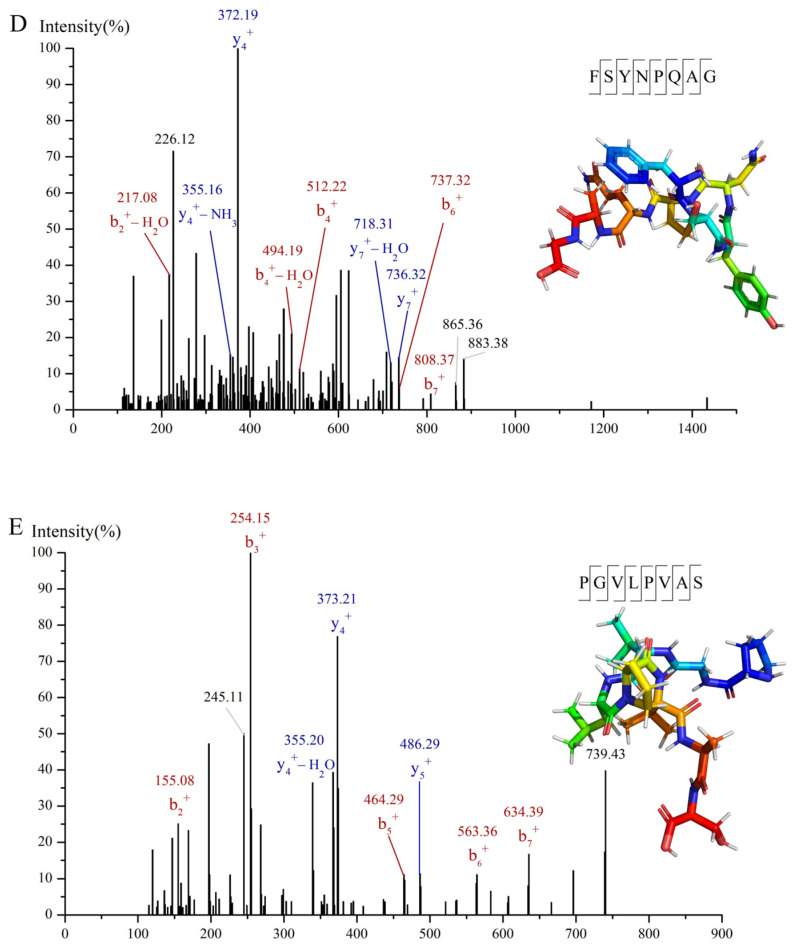
Total ion chromatogram of C-I (**A**) and MS/MS spectrum of FFVPPSQQ (**B**), FYNPAAGR (**C**), FSYNPQAG (**D**), and PGVLPVAS (**E**).

In order to identify the peptides with α-glucosidase inhibitory activity in the isolated components, the amino acid sequences of C-I were identified by nano-HPLC-MS/MS and a total of 643 peptides were obtained (Figure 3A). The promising peptides were further screened according to the molecular weight, amino acid composition, and binding energy with α-glucosidase. According to previous studies, peptides with strong α-glucosidase inhibitory activity were short peptides with a relative molecular weight below 1000 Da [39], due to the fact that lower molecular weight peptides could enter the active site of α-glucosidase to bind to α-glucosidase. However, VTGRFAGHPAAQ (1211 Da) from egg yolk protein hydrolysates also exhibited strong α-glucosidase inhibitory activity [40]. Therefore, molecular weight less than 1300 was set as the first screen condition. On the other hand, amino acid composition contributed a lot to the α-glucosidase inhibition. α-glucosidase inhibitory peptides were generally accompanied by a high degree of hydrophobicity [9,41]. Arg at the C-terminal was one of the features of potent α-glucosidase inhibitory peptides [7]. Therefore, peptides with hydrophobic amino acid residues and Arg at the C-terminal were screened. A total of 23 out of 643 peptides were screened and conducted further virtual screening to select the most potent peptides. The data of 23 peptides are displayed in Table 1.

The binding energy of peptides and α-glucosidase were predicted using molecular docking. 2QMJ was chosen as the crystal structure of α-glucosidase because of the homo sapiens attribute and the high resolution of 1.9 Å. The docking of acarbose and α-glucosidase was produced firstly to validate the docking method. It could be observed that acarbose bonded to residues Asp203, Thr205, Asp327, Arg526, Asp542, and His600 of 2QMJ and eight hydrogen bonds were formed. The binding sites were the same as those of a previous study [7]. The binding energy was −7.6 kcal/mol, similar to −7.63 kcal/mol given by Wang et al. [42]. These results indicate the credibility of the docking method. The lower the binding energy required for peptides to bind to α-glucosidase, the easier it is to inhibit the activity of α-glucosidase. Therefore, the binding energy lower than −7.6 kcal/mol was set as another screening condition. Four peptides (FYNPAAGR, PGVLPVAS, FFVPPSQQ and FSYNPQAG) with potential α-glucosidase inhibitory activity were screened. PGVLPVAS showed the lowest binding energy, followed by FYNPAAGR, FFVPPSQQ, and FSYNPQAG. Meanwhile, it was generally believed that the effective docking of small molecular ligands and receptors occurred if the RMSD value was less than 2. The RMSD values of the four peptides were all less than 2, suggesting the successful molecular docking. MS/MS spectrum of four peptides were shown in Figure 3B–E.

### 3.4. Analysis of Molecular Binding Mechanism

**Figure 4:**
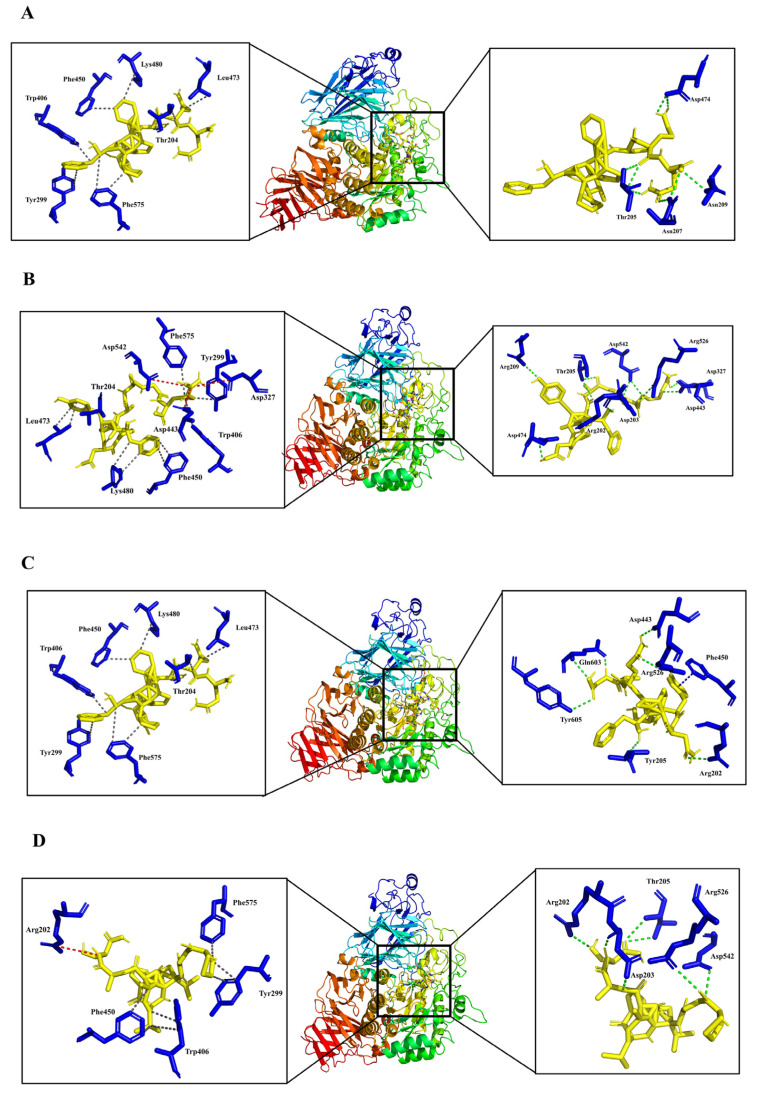
Docking results of peptides with α-glucosidase ((**A**) FFVPPSQQ, (**B**) FYNPAAGR, (**C**) FSYNPQAG, (**D**) PGVLPVAS). In the enlarged image, the yellow bar model represent polypeptides and the blue bar model represent amino acid residues. Green and grey dashed lines represent hydrogen bonding and hydrophobic interactions. Salt bridges and π-stacking are represented by red and blue dashed lines.

The active site of the α-glucosidase applied in this study is a pocket formed by four parts, namely C-terminal β-strand residues originating from the (β/α) _8_-barrel structure (residues 270–651), a loop belonging to the N-terminal domain (residues 200–217), and parts of catalytic Insert 1 (residues 367–416) and Insert 2 (residues 447–492). Asp443 and Asp542 play crucial roles in α-glucosidase catalytic reactions [43]. It was suggested that if residues in the active site were occupied by inhibitors, an apparent steric effect would happen, thus hindering the formation of the enzyme-substrate complex and the break of the glycosidic bond [41]. As shown in Figure 4, all four peptides bond to the active site of α-glucosidase, suggesting inhibitory activity. FYNPAAGR was observed to bind the maximum residues in the active site, followed by FSYNPQAG, PGVLPVAS, and FFVPPSQQ. Similarly, FYNPAAGR showed the highest number of interactions with 11 hydrogen bonds with Arg202, Asp203, Thr205, Asn209, Asp327, Asp443, Asp474, Arg526, Asp542, 10 hydrophobic interactions with Thr204, Tyr299, Trp406, Phe450, Leu473, Lys480, and Phe575, and 3 salt bridges with Asp327, Asp443, and Asp542. The binding mode of PGVLPVAS to α-glucosidase residues was similar to that of FYNPAAGR, with seven hydrophobic interactions with Tyr299, Trp406, Phe450, and Phe575, seven hydrogen bonds with Arg202, Asp203, Thr205, Arg526, and Asp542, and one salt bridges with Arg202. Only nine hydrophobic interactions and eight hydrogen bonds were found in the action of FFVPPSQQ and α-glucosidase. In particular, FSYNPQAG was found to form one π-stacking with Phe450, in addition to seven hydrogen bonds and six hydrophobic interactions with α-glucosidase. The molecular docking results indicated that most of the interactions between peptides and α-glucosidase residues were hydrogen bonds and hydrophobic interactions, thus contributing to the potential α-glucosidase inhibitory activity. The results were in accordance with previous studies which demonstrated that the interaction between α-glucosidase inhibitory peptides and α-glucosidase was mainly hydrogen bonding, hydrophobic interactions, and salt bridges [44], resulting in peptides with potential α-glucosidase inhibitory activity.

### 3.5. Chemical Stability of α-Glucosidase Inhibitory Peptides

**Figure 5:**
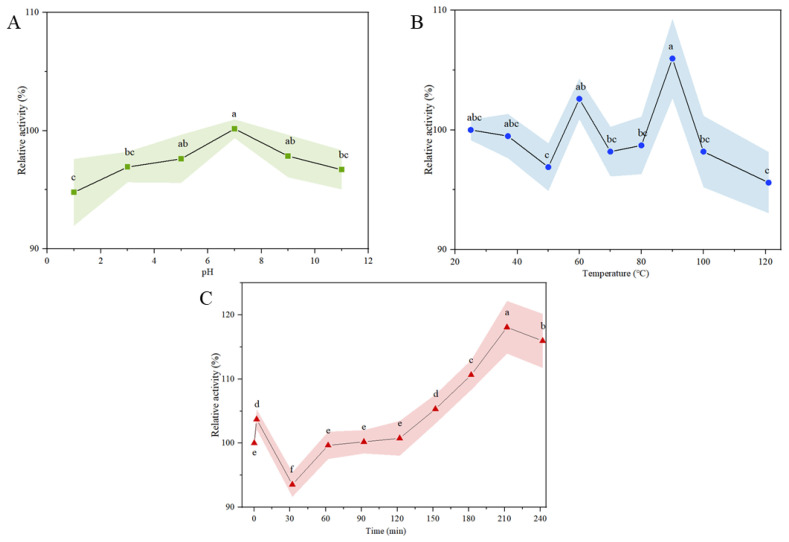
α-glucosidase inhibitory stability of PMHs against pH (**A**), hot (**B**), and in vitro gastrointestinal (**C**) with oral, gastric, and intestinal phases. Different letters indicate significant differences (*p* < 0.05).

Bioactive peptides will inevitably undergo processing, at variable pH and temperature, and gastrointestinal digestion before reaching the active pocket of α-glucosidase. So, it is practical to evaluate the bioactivity stability of PMHs under these conditions to ensure PMHs inhibit α-glucosidase in the body. As shown in Figure 5A, the relative activities of bioactive peptides at pH 1, 3, and pH 11 were significantly (*p* < 0.05) lower than other pH levels, which may be due to the inactivation of some fragments of bioactive peptides under extremely acidic and alkaline pH conditions [45]. Therefore, PMHs were suggested to be included in food at a pH between 4.0 and 10.0. The α-glucosidase inhibitory stability at different temperatures (25 °C to 121 °C) is shown in Figure 5B. The relative activity was more than 95% at all temperatures investigated, indicating PMHs had high thermal stability. Notably, the α-glucosidase inhibitory activity increased by 2.59% treated at 60 °C for 30 min and 5.97% treated at 90 °C for 30 min, which may be due to the conformation or active amino acid residue changes during thermal treatment [25]. The relative activity of 95.59 ± 2.56% at 121 °C for 30 min was the lowest. Overall, the α-glucosidase inhibitory peptide in PMHs had good hot stability and could be applied in hot processing.

According to Brodkorb et al. [26], although proteins are not affected by oral salivary enzymes, oral digestion must be included in all simulated food digestion processes. Therefore, the INFOGEST model was used to investigate the in vitro digestion stability of α-glucosidase inhibitory peptides in PMHs. As shown in Figure 5C, the relative inhibition rate of the sample increased to 103.28% after simulated oral digestion. The reason was unknown. After intragastric digestion, the relative inhibitory activity of bioactive peptides decreased slightly at 32 min and then increased gradually. The relative inhibitory activity increases gradually during intestinal digestion, which may be due to the exposure of more sites binding to α-glucosidase. Similar results were reported in camel casein hydrolysates and bovine casein hydrolysates the IC_50_ of which decreased significantly after simulated gastrointestinal digestion [41]. These results indicated that gastrointestinal digestion would not lessen the α-glucosidase inhibitory activity of PMHs, which was critical for PMHs to exert the health-beneficial effect.

## 4. Conclusions

In this study, four types of novel α-glucosidase inhibitory peptides, FYNPAAGR, PGVLPVAS, FFVPPSQQ, and FSYNPQAG, were identified by virtual screening from hot-pressed peanut meal protein hydrolysates. The molecular docking results indicated that four interactions (hydrogen bonding, hydrophobic interaction, salt bridges, and π-stacking) formed between peptides and α-glucosidase. Hydrogen bonding and hydrophobic interaction played significant roles. Moreover, the α-glucosidase inhibitory peptides of PMHs remained stable after pH treatment, heat treatment, and in vitro gastrointestinal digestion. Our results not only shed light on current knowledge about hypoglycemic peptides but also provide an option for value-added processing of hot-pressed peanut meals.

## Figures and Tables

**Table 1:** Basic information on the 23 peptides screened from C-I.

Sequence	Molecular Weight (Da)	Binding Energy (kcal/mol)
MVDVT	475.24	−6.7
PGVLPVAS	739.43	−8.6
VEPTPVQ	769.41	−6.3
LAFPGSGE	777.38	−7.3
VIQQGQAT	844.45	−6.5
ALRIPSGF	860.49	−6.6
FSYNPQAG	883.39	−7.7
FYNPAAGR	895.44	−8.1
GALDPVQVT	899.48	−5.3
VQVVDSNGN	931.45	−5.2
TATDLVLTV	932.52	−7.0
FFVPPSQQ	949.47	−8.0
AEYTAPVAE	950.44	−6.8
GDITNPINL	956.50	−5.3
TISPLPVIK	967.61	−6.7
ISPLPVIKE	995.57	−6.8
LDLVPTLPQ	995.61	−6.5
VNELDLPILG	1082.61	−6.3
ADAYAATGLLQ	1093.55	−6.0
LKNNNPFKF	1121.61	−5.1
IAVGDVIPDGTL	1169.64	−4.4
IYNPQAGSLKT	1191.64	−4.8
NTGSPITVPVGR	1197.66	−4.8

## Data Availability

Data are available when requested.
